# Interaction Between Odor Identification Deficit and APOE4 Predicts 6-Year Cognitive Decline in Elderly Individuals

**DOI:** 10.1007/s10519-019-09980-9

**Published:** 2019-11-23

**Authors:** Jonas K. Olofsson, Maria Larsson, Catalina Roa, Donald A. Wilson, Erika Jonsson Laukka

**Affiliations:** 1 Department of Psychology, Stockholm University, Frescati Hagväg 9A, 11419 Stockholm, Sweden; 2 Child Study Center, Child and Adolescent Psychiatry, New York University School of Medicine, New York, USA; 3 Emotional Brain Institute, Nathan Kline Institute, Orangeburg, USA; 4 Aging Research Center, Karolinska Institutet and Stockholm University, Stockholm, Sweden; 5 Stockholm Gerontology Research Center, Stockholm, Sweden

**Keywords:** Dementia, Alzheimer disease, Olfactory perception, Memory, Aging, Mild cognitive impairment

## Abstract

Olfactory identification impairment might indicate future cognitive decline in elderly individuals. An unresolved question is to what extent this effect is dependent on the ApoE-*ε*4, a genotype associated with risk of Alzheimer’s Disease (AD). Given the current concern about reproducibility in empirical research, we assessed this issue in a large sample (*n* = 1637) of older adults (60 – 96 years) from the population-based longitudinal Swedish National Study on Aging and Care in Kungsholmen (SNAC-K). A hierarchical regression analysis was carried out to determine if a low score on an odor identification test, and the presence of ApoE-*ε*4, would predict the magnitude of a prospective 6-year change in the Mini-Mental State Examination (MMSE) after controlling for demographic, health-related, and cognitive variables. We found that overall, lower odor identification performance was predictive of cognitive decline, and, as hypothesized, we found that the effect was most pronounced among ApoE-*ε*4 carriers. Our results from this high-powered sample suggest that in elderly carriers of the ApoE-*ε*4 allele, odor identification impairment provides an indication of future cognitive decline, which has relevance for the prognosis of AD.

## Introduction

Old age is often accompanied by a decline in sensory capabilities (Frenck et al. 1998; Murphy et al. 2002; Watabe-Rudolph et al. 2011). Exaggerated sensory decline trajectories are recognized as early behavioral markers for global cognitive impairment (Albers et al. 2015), which in turn is a well-established pre-clinical manifestation of dementia caused by neurodegenerative disease (Bäckman et al. 2005; Thorvaldsson et al. 2011). In particular, olfactory dysfunction has been suggested to indicate neurodegenerative disease progression in mesolimbic brain areas (Wilson et al. 2007; Devanand et al. 2008). Several research overviews have highlighted the severe olfactory loss observed in patients with Alzheimers’ and Pakinsons’ disease (Mesholam et al. 1998; Murphy 2019; Rahayel et al. 2012; Welge-Lüssen 2009). Olfactory dysfunction is a more robust indicator than auditory or visual impairment of future mild cognitive impairment (MacDonald et al. 2018). Olfactory dysfunction, whether assessed objectively or by self-report, is consistently found to predict future dementia and increased mortality risk (Ekström et al., 2017, 2019; Stanciu et al. 2014; Devanand et al. 2000; Devanand et al. 2015a, b). The parallel decline of visual-based cognitive abilities and olfactory capabilities in early-stage dementia might be due to atrophy in regions such as parahippocampal and orbitofrontal cortex that process both olfactory and multisensory information (Devanand et al. 2008; Dintica et al. 2019).

Olfactory dysfunction is common among healthy old individuals, with prevalence ranging between 15 and 70% depending on sample characteristics and diagnostic criteria (Seubert et al. 2017; Murphy et al., 2002; Schubert et al., 2012). As olfactory impairments are common, a key challenge is to localize subgroups where such impairments may be especially informative with regard to future cognitive development. Apolipoprotein E *ε*4 carriers may constitute such a subgroup. The ApoE gene is believed to be involved in the regenerative process of neurons, and is expressed in the olfactory bulb and the olfactory epithelium (Nathan et al. 2005; Struble et al. 1999), which are also the areas where the renewal of olfactory cells takes place (Watabe-Rudolph et al. 2011). ApoE has three allelic variations (*ε*2, *ε*3, *ε*4). The *ε*4 variant has been linked to olfactory dysfunction in old age in several independent studies (Graves et al. 1999; Olofsson et al. 2009, 2010) and identified as a risk factor for neurodegenerative diseases such as Alzheimer’s Disease (AD) (Corder et al. 1993), as well as non-pathological cognitive decline in healthy older adults (Bondi et al. 1995; Henderson et al. 1995). Recent findings suggest that carriers of the ApoE-*ε*4 allele may develop olfactory deficits along with episodic memory deficits already in middle-age (Josefsson et al. 2017).

Olofsson et al. (2009) reported that deficits in olfactory identification at baseline could dissociate between norm-level and exacerbated decline in global cognitive performance across a 5-year follow-up period in a sample of 501 cognitively intact older adults from the Betula study conducted in Umeå, Sweden (Nilsson et al. 1997, 2004). Interactive effects were found between odor identification, age, and the genotype ApoE-*ε*4, suggesting that elderly carriers of the *ε*4 allele that also performed poorly on an odor identification assessment, displayed an overall cognitive decline that was about twice as large as that of participants who exhibited only one, or neither, of these risk factors. These results are consistent with past research reporting that impaired olfactory performance was predictive of impending cognitive decline (Graves et al. 1999; Swan and Carmelli 2002). However, not all reports show an interaction effect between olfactory loss and the *ε*4 allele in predicting cognitive decline rates (Dintica et al. 2019; Devanand et al. 2015a, b). This suggests that more studies are needed on high-powered samples before this interaction is confirmed.

Here, we sought to reproduce the main finding from our prior work (Olofsson et al. 2009) in a much larger sample provided by the SNAC-K project in Stockholm, Sweden. We hypothesized that baseline olfactory performance would predict cognitive decline within a follow-up interval of six years (2001–2007) and that the effect would be primarily observed in ApoE-*ε*4 carriers. The methods used in the current study were modelled after our prior work using data from the Betula study in Umeå, Sweden (Olofsson et al. 2009), to maximize the similarity between the studies. A successful replication in a different population-based study cohort would strengthen the generalizability of previous findings, and provide further support for the notion that poor odor identification ability is a reliable marker of future cognitive decline, especially among older carriers of the ApoE-*ε*4 allele (Graves et al. 1999; Olofsson et al. 2009).

## Method

### Participants

The data used in the study were derived from the Swedish National Study on Aging and Care in Kungsholmen (SNAC-K). SNAC-K is a longitudinal population-based study on aging and health that started in 2001. The original study population comprised of 4590 people randomly selected from the Kungsholmen area in central Stockholm, Sweden. At baseline, 3363 participants were assessed, belonging to 11 pre-specified age cohorts: 60, 66, 72, 78, 81, 84, 87, 90, 93, 96, and 99 years and older. The examination lasted approximately 6 h and involved a social interview and assessment of physical functioning (performed by nurses); a clinical examination including geriatric, neurological, and psychiatric assessment (performed by physicians); and neuropsychological testing (performed by psychologists). Older participants (≥ 78 years at baseline) are called back for re-assessments every 3 years and younger participants every 6 years. Of 2848 participants who completed the neuropsychological test battery at baseline (Laukka et al. 2013), 2569 provided data on the olfactory test. Reasons for dropout or exclusion of data (*n* = 279) included self-reported anosmia (*n* = 95, 34.1%), olfactory over-sensitivity (*n* = 17, 6.1%), asthma or allergies (*n* = 48, 17.2%), tiredness (*n* = 31, 11.1%), refusals (*n* = 36, 12.9%), or other reasons (*n* = 52, 18.6%).

**Fig. 1:**
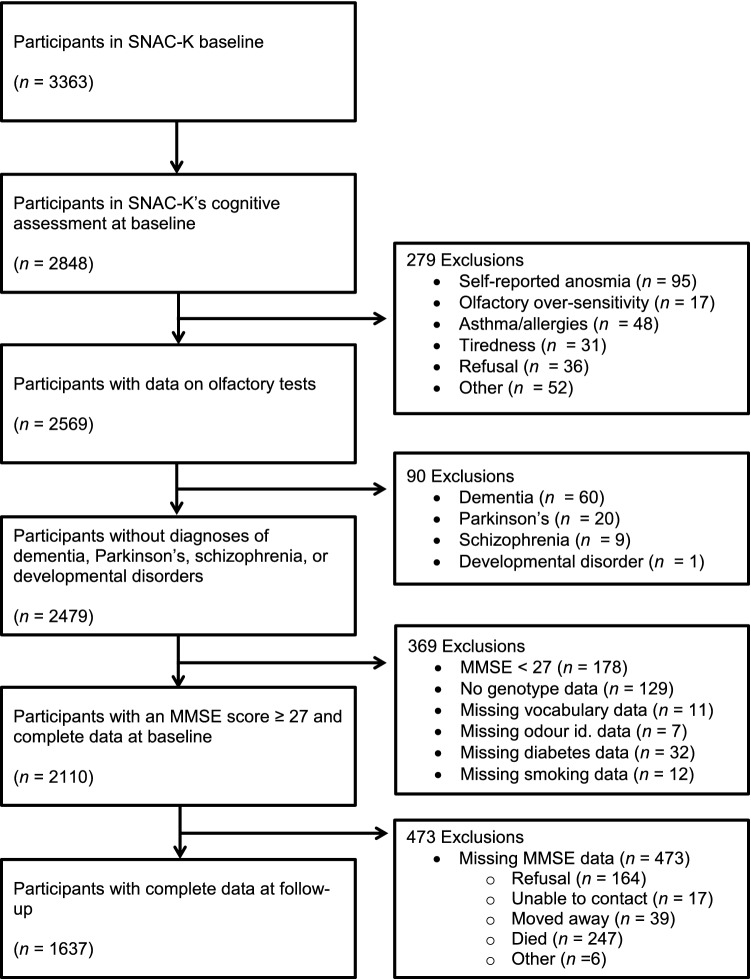
Exclusion flowchart

To retain a sample of participants with relatively intact cognitive abilities, additional exclusion criteria were applied to exclude participants with a history of schizophrenia or developmental disorder, and those who were diagnosed with dementia (DSM IV criteria), or Parkinsons’ disease (CERAD criteria) at the baseline assessment. Furthermore, participants with a MMSE score below 27 at baseline were excluded. Though Olofsson et al.’ (2009) original study used an MMSE cut-off score of 25 and above, more recent research has suggested 27 as an optimal cut-off score for screening for Mild Cognitive Impairment (MCI) (Tsai et al. 2016). We decided that the more conservative criterion of MMSE 27 or above would provide the optimal selection of cognitively intact participants, although we also conducted follow-up analyses using the previous cut-off value. The cut-off score of 27 was only applied at baseline and not at follow-up, in order to avoid restricting the range of cognitive decline that provided our outcome measure. Furthermore, participants who developed neurodegenerative disorders during the follow-up interval were not excluded or otherwise controlled for in the analysis, as this is a common cause of age-related cognitive decline. After applying all exclusion criteria, there were 2110 participants with complete data at baseline (see Fig. 1 for specifics). At the six-year follow-up, 1637 participants were re-assessed with the MMSE (see Table 1 for descriptive characteristics of the study sample). Participants who did not return for follow-up testing tended to be older, less educated, have higher cardiovascular disease burden, and score lower on all cognitive tasks at baseline (including the olfactory task; *p*s < 0.05). They were also more likely to have diabetes, be current smokers, or have a history of cerebrovascular disorders at baseline (*p*s < 0.05). In contrast, there were no differences with regard to sex, *APOE* genotype, or history of head trauma (*p*s > 0.05).

**Table 1:** Participant characteristics (percentage in parentheses; *n* = 1637)

Age in years, mean ± SD	69.83 ± 8.66
Gender, *n* males/females	624/1013
Education in years, mean ± SD	12.79 ± 4.19
ApoE-*ε*2, *n*	233 (14.2)
ApoE-*ε*4, *n*	488 (29.8)
Diabetes, *n*	120 (7.3)
Head injury, *n*	224 (13.7)
Cardiovascular disease burden, mean ± SD	0.29 ± 0.61
Smoking, *n*	234 (14.3)
Cerebrovascular disease, *n*	105 (6.4)
Odor identification, proportion correct mean ± SD	0.77 ± 0.17
Vocabulary test, mean ± SD	23.73 ± 4.35
MMSE at baseline, mean ± SD	29.27 ± 0.83
MMSE at 6-year follow-up, mean ± SD	27.92 ± 2.82

All parts of SNAC-K have been approved by the Karolinska Institutet Ethical committee (dnr 01-114) or the Regional Ethical Review Board (dnrs 04-929/3, Ö 26-2007). The research in SNAC-K was conducted according to the ethical standards stated in the 1964 Declaration of Helsinki. All participants directly provided informed written consent, or in cases where the participants were too severely cognitively impaired, the consent was provided by their next-of-kin.

### Materials and procedure

#### Odor identification

As part of a comprehensive cognitive testing battery, the Sniffin’ TOM odor memory test, a standardized assessment with high test–retest reliability, was used to assess odor identification performance (Croy et al. 2015; Hummel et al. 1997). Odor identification was assessed in conjunction with an initial odor recognition memory assessment. Sixteen odors were presented in felt-tip pens during 5 s odor exposure and 15 s interstimulus intervals: apple, banana, cinnamon, cloves, coffee, fish, garlic, leather, lemon, liquorice, mushroom, peppermint, petrol, pineapple, rose, and turpentine. Participants were instructed to freely identify the odor; if they did not provide a response, or responded incorrectly, they were presented with four response alternatives (cued identification). In the present context, a correct response under either response format (free or cued) was considered correct (Seubert et al. 2017). Test procedures have been described in more detail elsewhere (Larsson et al. 2016). This procedure is somewhat different from the Betula study, where no olfactory naming or recognition tasks were carried out (Larsson et al. 2004). Furthermore, and as is common in the field of olfactory research, the odors and response alternatives are different across studies. However, we assumed that the two studies would assess the same underlying ability to identify odors.

#### Cognitive variables

##### Global cognitive function

The Mini-Mental State Examination (MMSE) was used to measure global cognitive performance. The MMSE is widely used to assess cognitive function and detect cognitive impairment. The MMSE (maximum score = 30) assesses general cognitive performance in various domains, including arithmetic skills, episodic memory, orientation, and language. The test is divided in two sections; the first section requires vocal responses to items assessing orientation, memory, and attention. The second section assesses the ability of the individual to follow verbal and written commands, write down sentences, and draw a replica of a displayed complex polygon (Folstein et al. 1975).

##### Vocabulary

As in our prior work (Olofsson et al. 2009), we statistically controlled for individual differences in vocabulary, as it may influence odor identification performance (Larsson et al. 2004). Thus, part 1 of the Synonyms Reasoning Blocks (SRB:1) (Dureman 1960) test was included. SRB comprises a 30-item multiple-choice vocabulary test to assess verbal knowledge. The participants are instructed to select a synonym for a target word out of five alternatives. The score is calculated by aggregating the number of correct synonyms achieved within the time limit of 7 min. Because the SRB has a similar format to the odor identification test (both assessments involve matching stimuli to a list of written alternatives), it is useful as a non-olfactory control task (Hedner et al. 2010).

#### Demographic variables

Various demographic factors influence an individual’s general health and aging processes. Differences in lifestyle choices and in the quantity and quality of resources available to an individual promotes disparities in health, accelerating or attenuating age-related deficits (Mobley et al. 2014). We considered several demographic variables including age, gender (male, female), and education (number of years of formal schooling). The information was collected following standard protocols.

#### Health variables

Information regarding health variables was collected through self-report, clinical examinations; medication lists, laboratory data, and by accessing the computerised Stockholm inpatient register. The vascular and other health-related variables included diabetes, history of head trauma, smoking (dichotomised into “current smoking” and “no current smoking”), cerebrovascular disease, and cardiovascular disease burden (a composite measure including a history of heart failure, atrial fibrillation, and coronary heart disease). In contrast to Olofsson et al. (2009), information regarding Ear-Nose-and-Throat (ENT) disorders was not collected in SNAC-K. However, considering that ENT disorders had no influence on outcomes in the previous study, we had no reason to believe they would be relevant in this study.

#### Genetic variables

Genotype information for ApoE (rs7412, rs429358) was obtained from peripheral blood samples through standard methods. The genotyping of ApoE was conducted using the Matrix-Assisted Laser Desorption/Ionization Time-Of-Flight (MALDI-TOF) analysis on the Sequenom Mass Array platform at the Mutation Analysis Facility, Karolinska Institutet (Darki et al. 2012). The results produced by this procedure were in Hardy Weinberg equilibrium and had a successful genotyping rate of 97%. Participants were grouped as any *ε*4 carriers versus no carrier, and any *ε*2 carriers versus no carriers. The genotype proportions of the sample at follow-up (*n* = 1637) were: *ε*2/*ε*2, 1% (*n* = 12); *ε*2/*ε*3, 10% (*n* = 171); *ε*2/*ε*4, 3% (*n* = 50); *ε*3/*ε*3, 59% (*n* = 966); *ε*3/*ε*4, 24% (*n* = 392); *ε*4/*ε*4, 3% (*n* = 46). Following the same protocol as our previous work (Olofsson et al. 2009), the ApoE-*ε*2 variant was included in the analysis to control for its reported protective effect against dementia syndromes such as AD (Conejero-Goldberg et al. 2014; Corder et al. 1994).

### Statistical analysis

A hierarchical regression analysis was carried out to determine if odor identification performance was a reliable predictor for MMSE scores at the 6-year follow-up, when MMSE baseline scores, demographic, genetic, and health-related variables were controlled for. An interaction term between odor identification and ApoE-*ε*4 was created by mean-centering each score of odor identification and ApoE-*ε*4, and multiplying them with each other (Odor id. × ApoE-*ε*4). Following our previous statistical procedure, the interaction variables were mean-centred for all statistical analyses in order to avoid possible multicollinearity (Olofsson et al. 2009).

The order in which each variable was entered and divided into different blocks for the hierarchical analysis followed our previous method (Olofsson et al. 2009). MMSE performance at the 6-year follow-up was used as the criterion measure, while MMSE performance at baseline was entered in the first block to control for the participants’ initial cognitive ability. Thus, the predictors added after the first block effectively constitute predictors of MMSE change. Demographic variables (age, gender, and education) were entered in the second block, as these variables were expected to be associated with health and cognitive performance. This influence needs to be accounted for when estimating the true predictive power of odor identification. Genetic information regarding the ApoE gene (presence of allele *ε*2 and *ε*4) was entered in the third block, since genetic influence is susceptible to interference from social and environmental factors. To account for effects of poor health on cognitive and olfactory performance, health-related variables were entered in the fourth block: diabetes, head injury, cardiovascular disease burden, smoking, and cerebrovascular disease. Odor identification (odor-to-word matching) and a non-olfactory cognitive control assessment of vocabulary (word-to-word matching; SRB:1) were then entered in the fifth block, while the two-way interaction between odor identification and ApoE-*ε*4 was entered in the final block.

## Results

The intercorrelations among all variables were calculated using Pearson correlation coefficients (see Table 2). Although strong correlations among the predictor variables would suggest a possible multicollinearity issue, the observed correlations here were weak to moderate, ranging from *r* = − 0.31, *p* < 0.001 to *r* = 0.41, *p* < 0.001. Most predictor variables were correlated with MMSE at follow-up with the exception of gender, ApoE-*ε*2, occurrence of head injury, and current smoking. In addition, data screening indicated no multicollinearity among any of the predictor variables, with all tolerance values being above 0.10 and variance inflation factors (VIFs) below 10. The observed significant correlations among odor identification, ApoE-*ε*4, and MMSE at follow-up motivated further examination of the data through hierarchical regression analysis to determine if these associations were unique and independent from possible mediating effects of the other variables.

**Table 2:** Intercorrelations among variables

Variable	1	2	3	4	5	6	7	8	9	10	11	12	13	14
1. Age	–													
2. Gender	**0.10**	–												
3. Education	**− 0.31**	**− 0.09**	–											
4. ApoE-*ε*2	0.00	0.01	0.00	–										
5. ApoE-*ε*4	**0.05**	0.01	**−** 0.01	**0.07**	–									
6. Diabetes	0.04	**− 0.12**	**− 0.05**	**−**0.01	0.04	–								
7. Head injury	**− 0.07**	**− 0.11**	0.03	**−**0.02	0.01	**−** 0.02	–							
8. Cardiovascular disease burden	**0.28**	**− 0.09**	**− 0.09**	**−**0.04	**− 0.03**	0.15	**0.05**	–						
9. Smoking	**− 0.14**	0.01	0.03	**−**0.02	0.01	**−** 0.03	0.03	**− 0.07**	–					
10. Cerebrovascular disease	**0.17**	**−**0.02	**− 0.08**	**−**0.04	**− **0.00	0.01	**−** 0.01	**0.13**	**−**0.01	–				
11. Odor identification	**− 0.22**	**0.10**	**0.15**	0.02	**0.04**	**−** 0.07	**−** 0.04	**− 0.12**	0.03	**− 0.07**	–			
12. Vocabulary	**− 0.33**	0.00	**0.41**	0.00	**− 0.02**	**−** 0.04	0.03	**− 0.08**	**0.05**	**− 0.08**	**0.18**	–		
13. MMSE at baseline	**− 0.29**	0.00	**0.22**	0.01	**−**0.04	**− 0.04**	**−** 0.04	**− 0.13**	**0.05**	**− 0.08**	**0.17**	**0.17**	–	
14. MMSE at 6-year follow-up	−** 0.38**	0.02	**0.26**	0.01	**0.07**	−** 0.06**	0.01	−** 0.13**	0.01	−**0.21**	**0.22**	**0.22**	**0.29**	–

Correlations in bold are significant, *p* < .05

The results from the hierarchical regression analysis are presented in Table 3 and indicate that after statistical control of baseline MMSE performance, all following blocks included in the regression analysis significantly accounted for a unique and significant proportion of the variance in cognitive performance (MMSE at follow-up). MMSE performance at baseline initially accounted for 8.5% of the variance in cognitive performance (*p* < 0.001), and after the demographic block was included the model as a whole accounted for 19.7%. This indicates that demographic variables explained an additional 11.2% of the variance in the criterion measure (*p* < 0.001), which was the largest contribution to the model of all the different blocks. The following block containing genetic variables accounted for an additional 0.9% of the variation (*p* < 0.001. However, of the two variables, only ApoE-*ε*4 contributed reliably, suggesting that the presence of an *ε*4 allele had a significant negative influence on MMSE change. Health variables accounted for another 2.4% of the variance in cognitive performance (*p* < 0.001), specifically the smoking and cerebrovascular disease variables.

**Table 3:** Hierarchical regression analysis for predicting cognitive performance (MMSE score) at follow-up (*n* = 1637)

	*R*2Δ	*R*^2^	*β*	*p*
1. MMSE at baseline	0.085	0.085	0.292	0.000***
2. Demographic	0.112	0.197		
Age			− 0.289	0.000***
Gender (1 = m, 2 = f)			0.041	0.068
Education			0.137	0.000***
3. Genetic	0.009	0.206		
ApoE-*ε*4			− 0.092	0.000***
ApoE-*ε*2			0.005	0.806
4. Health	0.024	0.229		
Diabetes			− 0.037	0.093
Head injury			− 0.009	0.684
Cardiovascular disease burden			0.009	0.711
Smoking			− 0.050	0.023*
Cerebrovascular disease			− 0.143	0.000***
5. Sensory/cognitive tests	0.014	0.243		
Odor identification			0.056	0.016*
Vocabulary test			0.115	0.000***
6. Two-way interaction	0.003	0.246		
Odor id. × ApoE-*ε*4			0.054	0.013*

*MMSE* Mini Mental State Examination

*p < 0.05; **p < 0.01; ***p < 0.001

The block consisting of performance in sensory/cognitive tests accounted for 1.4% of the variance (*p* < 0.001), with both participants’ performance in odor identification and in the vocabulary test exerting a significant positive influence on cognitive ability at follow-up. This indicates that better odor identification performance and vocabulary test performance are associated with less changes in cognitive ability, even after controlling for the effects of MMSE at baseline, demographic, genetic, and health variables. The final block containing the two-way interaction between odor identification and ApoE-*ε*4 accounted for 0.3% of the variance (*p* < 0.05), indicating that the interaction between odor identification performance and ApoE-*ε*4 significantly influenced cognitive change.

**Fig. 2:**
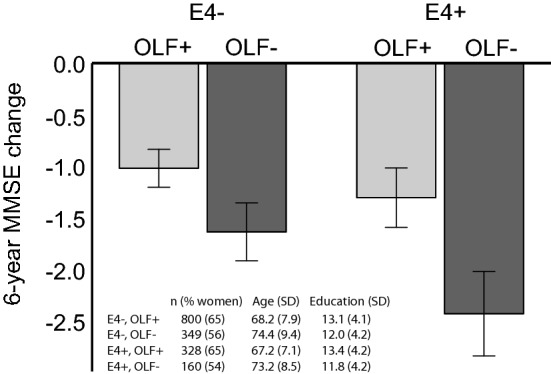
Mean change in MMSE scores (± 2SD) from baseline to follow-up in *ε*4 carriers (E4+) and non-carriers (E4−), divided into groups of odor identification scores below median (OLF−, light grey bars) or above median (OLF+, dark grey bars). Insert shows descriptive demographics (n, age, and education) in these four groups

Figure 2 illustrates the interaction between odor identification and ApoE-*ε*4, based on a median split of the odor identification scores which was applied in order to classify participants. Figure 2 uses data unadjusted for demographic differences between the groups, which are instead shown in a table insert. Among participants with below-median odor identification score, ApoE-*ε*4 carriers show a larger decrease in MMSE score. In participants with above-median odor identification score, the ApoE-*ε*4 effect is less notable. Note that in Fig. 2, demographic characteristics (which were accounted for in the statistical analysis) differ somewhat between groups of high and low odor identification performance, but not between ApoE-*ε*4 carriers and non-carriers. To strengthen the validity of this main result, we conducted two follow-up analyses. First, we assessed whether the interaction effect would be retained if we replaced odor identification with vocabulary in the interaction with ApoE-*ε*4. The regression was otherwise identical to that presented in Table III. The results showed that the vocabulary × ApoE-*ε*4 interaction was not significant (t = 1.580; p = 0.114), which further strengthened our conclusion that the observed interaction effect was olfactory-specific. Second, we conducted a follow-up analysis to assess whether the main result would be retained when using the more liberal inclusion criterion of MMSE 25 or above. Results from this slightly larger sample (n = 1689) were highly similar to those presented in Table 3; the interaction of odor identification × ApoE-*ε*4 was still significant (p = 0.014), supporting our main result.

## Discussion

As olfactory deficits emerge as a potential biomarker for cognitive impairment and dementia (Murphy 2019), high-powered studies are needed to determine subgroups where this relationship is especially strong. The present study uses a combination of a longitudinal study design, and assessments of cognition, olfaction and genetics, to enable conclusions regarding relationship among olfaction, ApoE, and 6-year cognitive decline. In line with previous research (Graves et al. 1999; Larsson et al. 2016; Olofsson et al. 2009), this investigation finds that the combination of poor olfactory identification and the ApoE-*ε*4 genotype is predictive of a future decline in cognitive performance, also after controlling for baseline cognitive function, demographic, genetic, and health factors. However, in line with other published reports (Devanand et al. 2015a, b; Schubert et al. 2008; Wilson et al. 2007), the present study also found that poor baseline olfactory identification alone predicted future cognitive decline (i.e. the effect of olfactory impairment on future cognitive decline is also present, albeit less pronounced, in ApoE-*ε*4 non-carriers). These results contribute to the growing body of research suggesting that olfactory identification deficits are useful in predicting cognitive decline. As the neural processes underlying successful odor identification are becoming more well established, in both rats and humans, this might provide an opportunity for cross-species translation of findings (Olofsson et al. 2019; Zhou et al. 2019).

The interaction effect between poor odor identification ability and ApoE-*ε*4 in predicting future cognitive decline fits well with what is known about regional accumulation of AD neuropathology in preclinical stages. According to the Braak staging model of AD, amyloid and tau pathologies are initiated in the entorhinal cortex and surrounding areas of the antero-medial temporal lobes, where olfactory processing takes place (Braak and Braak 1995; Braak et al. 1993). At this stage, such focal pathology is yet to affect global cognitive functions. Odor identification assessments might provide an index of the severity of a neuropathological load specifically in mediotemporal regions, which are affected in preclinical stages of AD (Devanand et al. 2008; Dintica et al. 2019; Hagemeier et al. 2016; Vassilaki et al. 2017). Why is the relationship between current olfactory impairments and future cognitive decline weaker in ApoE-*ε*4 non-carriers? This might be a result of different patterns of cortical atrophy, as those who develop AD without ApoE-*ε*4 display cortical atrophy predominantly in fronto-parietal cortex, which is accompanied by executive function deficits, whereas AD patients with ApoE-*ε*4 display cortical atrophy predominantly in the mediotemporal cortex, which is accompanied by memory deficits (Wolk et al. 2010). As fronto-parietal regions are less critical for olfaction, cognitive functions may thus decline in some non-carriers, largely independently of their olfactory abilities. However, it should be noted that other biological pathways are also possible to explain the effects of ApoE-*ε*4. For example, mice implanted with human ApoE-*ε*4 show an enhanced response to odors in the olfactory bulb and piriform cortex, as well as a lack of olfactory habituation across multiple stimulations (East et al. 2018). This finding suggests a neuronal inhibition failure, perhaps explained by deficits in the exosomal pathways of neurons (Peng et al. 2017). Given these observations in mice, which lack typical AD neuropathology, it is possible that olfactory impairments in human ApoE-*ε*4 carriers may be caused also by non-pathological mechanisms such as exosomal changes.

Our current focus on replicating our prior findings is in line with the increasing focus on reproducible research. Unfortunately, while reproducibility is considered a cornerstone of empirical science, a large body of work suggests many empirical research findings are exaggerated or false (Ioannidis 2005; Open Science Collaboration 2015; Prinz et al. 2011). It has been estimated that a successful replication of a prior positive finding increases the likelihood of the finding being true from 50 to 95% (Dreber et al., 2015). Indeed, our successful replication of the previously published interaction effect (Olofsson et al. 2009), using a sample three times larger, suggests the effect is robust. However, because of the small effect size it might only be observed in high-powered samples. Our observed interaction between olfactory impairment and ApoE-*ε*4 was not observed in all prior studies (Devanand et al. 2015a, b; Dintica et al. 2019). We suggest that this discrepancy may be due to the fact that *ε*4 is rare, its effects on olfaction are overall of small size, and its relationship with AD is especially strong in individuals with European and Asian ancestry, and weaker in individuals with African ancestry (Farrer et al. 1997; Rajabli et al. 2018). While the latter finding might be due to a variety of mechanisms, recent results indicate an important role of ancestry-specific genetic effects near the ApoE region in the genome, rather than non-genetic cultural or environmental factors (Rajabli et al. 2018). We speculate that the studies conducted at the Rush Memory and Aging Project (Dintica et al. 2019), Washington Heights/Inwood Columbia Aging Project (Devanand et al. 2015a, b), or the Swedish Adoption/Twin Study of Aging (Finkel et al. 2011), including samples that are different in demographics and size compared the Swedish Betula and SNAC-K studies, would be less likely to observe an interaction between olfactory impairment and ApoE-*ε*4, whereas the present study, using a large sample of Swedish participants, might be more likely to observe an interaction. We recommend that future empirical work on olfaction and cognition/AD outcomes report interactions with ApoE, even when negative, so that meta-analyses may answer such questions more definitively.

The present work highlights the role of sensory deficits as a complement to cognitive decline as a means of early-stage detection of AD and other age-related disorders (Bacon et al. 1998). Olfaction might provide the best sensory target for AD prognosis, although sensory markers focusing on the visual and auditory systems are also viable (Murphy 2019). A critical issue for future research is to compare different olfactory tests in their prediction of cognitive decline. Different olfactory abilities may diminish at different rates with age (Larsson et al. 2016). Meta-analytic evidence suggests that “cognitive” olfactory functions (identification, recognition) may show larger impairments in Alzheimers disease compared to “sensory” olfactory functions (detection, discrimination) (Rahayel et al. 2012). Importantly, neuropathology can manifest in brain regions involved in olfactory functions without affecting other sensory areas (Van Hoesen and Solodkin 1994). Given that current olfactory assessments are not optimized for the detection of specific conditions, the development of such assessments is likely to provide stronger effects and better diagnostic utility.

Among the possible limitations of this study is the use of MMSE to assess cognitive ability. Though MMSE is widely used, the MMSE has been criticized for its psychometric properties (Mitchell 2013; Proust-Lima et al. 2007; Tombaugh and McIntyre 1992). Despite its limitations, MMSE remains a valuable tool for assessing overall cognitive function (Han et al. 2000) and the use of MMSE change scores in the present work was justified by our aim to replicate the methods used in our previous work (Olofsson et al. 2009). In future studies, we will compare decline trajectories in multiple cognitive domains and across multiple time points (Josefsson et al. 2017). Such data will allow us to better account for measurement error and establish decline trajectories (Singer and Willett 2003). Another limitation of our work is that 23% of participants did not return for the follow-up assessment, mostly because poor health and incident death was prevalent in this sample of older adults. We analysed differences between those who returned and those who did not return, and found the latter group to be older, of poorer health and performing more poorly on cognitive tests (including the olfactory assessment) at baseline. However, ApoE status did not affect risk of dropout, and as olfactory dysfunction was only linked to dropout as part of an overall pattern of poor cognitive performance, it seems unlikely that our interaction effect was somehow driven by dropout. Finally, we note that odor identification was somewhat less effective than vocabulary in predicting cognitive impairment. We speculate that this advantage of vocabulary over olfaction was due to two reasons. First, the olfactory test was brief, including 16-items compared to the 30-item vocabulary test, and adding more items to the odor identification test would likely lead to higher reliability and predictive accuracy (Freiherr et al. 2012). Second, vocabulary is often used as a proxy for lifestyle factors that enhances “cognitive reserve” capacity and prevents cognitive decline (Soldan et al. 2017). While it is thus not surprising that vocabulary effectively predicts cognitive decline, future studies should include more in-depth olfactory assessments in order to optimize its role as a complement to other cognitive assessments.

To conclude, the present findings replicate and extend previous work by showing that both odor identification performance at baseline, and the interaction of odor identification performance and ApoE-*ε*4, can be used to predict the magnitude of future cognitive decline. These findings are promising as they have clinical implications for the efforts to distinguish between normative and harmful cognitive decline trajectories in old age, and for the early identification of individuals at risk of dementia conversion.
